# Comparing Molecular Dynamics Force Fields in the Essential Subspace

**DOI:** 10.1371/journal.pone.0121114

**Published:** 2015-03-26

**Authors:** Fernando Martín-García, Elena Papaleo, Paulino Gomez-Puertas, Wouter Boomsma, Kresten Lindorff-Larsen

**Affiliations:** 1 Molecular Modelling Group, Centro de Biología Molecular Severo Ochoa (CSIC-UAM), C/Nicolás Cabrera 1, Cantoblanco, Madrid, Spain; 2 Biomol-Informatics SL, Parque Científico de Madrid, Cantoblanco, Madrid, Spain; 3 Structural Biology and NMR Laboratory, Department of Biology, University of Copenhagen, Copenhagen, Denmark; Jacobs University Bremen, GERMANY

## Abstract

The continued development and utility of molecular dynamics simulations requires improvements in both the physical models used (force fields) and in our ability to sample the Boltzmann distribution of these models. Recent developments in both areas have made available multi-microsecond simulations of two proteins, ubiquitin and Protein G, using a number of different force fields. Although these force fields mostly share a common mathematical form, they differ in their parameters and in the philosophy by which these were derived, and previous analyses showed varying levels of agreement with experimental NMR data. To complement the comparison to experiments, we have performed a structural analysis of and comparison between these simulations, thereby providing insight into the relationship between force-field parameterization, the resulting ensemble of conformations and the agreement with experiments. In particular, our results show that, at a coarse level, many of the motional properties are preserved across several, though not all, force fields. At a finer level of detail, however, there are distinct differences in both the structure and dynamics of the two proteins, which can, together with comparison with experimental data, help to select force fields for simulations of proteins. A noteworthy observation is that force fields that have been reparameterized and improved to provide a more accurate energetic description of the balance between helical and coil structures are difficult to distinguish from their “unbalanced” counterparts in these simulations. This observation implies that simulations of stable, folded proteins, even those reaching 10 microseconds in length, may provide relatively little information that can be used to modify torsion parameters to achieve an accurate balance between different secondary structural elements.

## Introduction

Molecular dynamics (MD) simulation is a well-established computational method that can be used to describe the dynamical properties of proteins and other macromolecules and to provide structural interpretations of experimental data [1,2]. Although MD simulations have already been used to provide a wealth of biophysical and biological insight, the continued utility of the method to help solve problems of increasing complexity is limited by two factors. First, the *precision* by which quantities can be estimated from simulations is inherently limited by our ability to sample sufficiently the conformational space accessible to the molecules being studied. In particular, only by averaging over a sufficient number of independent conformations can the statistical fluctuations that are inherent in MD simulations be averaged out to provide robust quantitative estimates. Second, the *accuracy* of these estimates depends crucially on the molecular mechanics force fields that are used to generate conformations in MD simulations. Sufficient accuracy is best quantified by comparison with experiments. For such comparisons to provide meaningful insight into any remaining force field deficiencies the simulations must, however, be sufficiently converged (i.e. estimates must be relatively precise) before deviations between experiments and simulations can uniquely be ascribed to the force field [3].

Based in part on continued developments in our ability to sample conformations, recent years have seen several important developments in molecular mechanics force fields. Using the ANTON computer, 10-microsecond simulations have previously been performed of two small proteins, ubiquitin (Ubq) and the B3 domain of Protein G (GB3), using eight different protein force fields and the TIP3P water model [4], and with the resulting ensembles compared to a broad range of experimental data. That study, together with complementary studies that focused in part on different force fields and sources of experimental data [5–7], have demonstrated that several of these force fields result in relatively accurate predictions of experimentally-derived NMR parameters including residual dipolar couplings, scalar couplings, relaxation order parameters and nuclear Overhauser enhancements [4,5,7,8]. The comparison to such NMR-derived parameters provides sensitive probes to validate the structure and dynamics described by MD simulations. In particular, among the eight force fields that were evaluated using the 10-microsecond simulations, the comparison of the simulations with the NMR data on Ubq and GB3 [4] determined that, in these tests, three different levels of agreement between simulation and experiment could be identified. (i) Four force fields (CHARMM22*, CHARMM27, Amber ff99SB-ILDN, and Amber ff99SB*-ILDN) all resulted in a reasonably good agreement between experiment and simulation. (ii) Two related force fields (Amber ff03 and Amber ff03*) resulted in an intermediate level of agreement with the experimental data. Finally, (iii) two force fields (OPLS and CHARMM22) resulted in reasonable agreement with experiments on short timescales, but a substantial conformational drift in the simulations resulted in a decreased agreement when the entire simulations were compared to the experiments. Together with similar comparisons with experimental data for short, flexible peptides, and the ability of the force fields to fold proteins to their native states, these studies afforded a systematic evaluation of several commonly employed force fields [4]. The results provided evidence for the continued improved accuracy of force fields, and in several cases these improvements were due to relatively minor changes in the torsion potential for the polypeptide backbone and side chains. Studies such as those described above, together with other considerations such as software implementations and the availability of parameters for molecules other than proteins, can be very useful e.g. when designing simulation studies on the relationship between protein structure and function or when studying biophysical properties of proteins and peptides.

As a complementary approach to evaluate the effect of the choice of force fields in MD simulations one may also compare the structural ensembles directly, by quantifying the overlap between the conformational spaces described by different ensembles. In this context, Principal Component Analysis (PCA) provides a straightforward and robust approach to analyse the structural space sampled in MD simulations [9,10]. For example, PCA has previously been employed to compare simulations with different force fields to an experimentally derived conformational ensemble [7], as well as to evaluate the sampling quality achieved in MD simulations [11–16].

Here we extend the previous analyses of10-microsecond long MD simulations of Ubq and GB3which focused on comparisons with experimental data [4] by an analysis of the ensembles aided by the use of PCA.These simulations are an order of magnitude longer than other simulations that have been analysed in this way, and provide a unique perspective of the similarities and differences between the eight different force fields, and an overall view of the effect the choice of force field can have on the structural ensemble obtained from MD simulations. We show that certain pairs of force fields (e.g. Amber ff99SB-ILDN and ff99SB*-ILDN), which differ substantially in their preferences for forming helical structures, result in structural ensembles that are essentially indistinguishable on the timescales explored here. This observation explains why these simulations give rise to very similar agreements with experiments, and also provides practical insight into the effect such force field changes will have on simulations of different kinds of systems. As a corollary, the results suggest that while simulations of these rather rigid proteins provide a very effective means to evaluate the accuracy of the description of the local structure of the proteins, larger conformational changes, such as those involving transitions between helix and coil structures, cannot be probed easily. We also find that simulations with Amber ff03 and ff03* force fields give rise to very similar ensembles that are distinctly different from those obtained with the ff99SB-derived force fields. Our results also show that, in several cases, the larger scale (loop) motions probed in simulations are rather well preserved across different force fields, prompting the suggestion that such “conserved” motions may robustly represent the structural dynamics of these proteins. At a finer level of detail, differences can be identified in the structures explored by the different simulations, and our analysis thereby complements the comparison with experiments in understanding the effect the choice of force field can have on a simulation. Finally, by analysing the timescales of motions explored in the reductive description of PCA we highlight the difficulty of obtaining converged simulations even of small, relatively rigid proteins.

## Methods

### Principal Component Analysis

PCA can be used to extract larger amplitude motions observed in MD trajectories through the eigenvectors (principal components, PCs) of the mass-weighted covariance matrix (*C*) of the atomic positional fluctuations [10]. We calculated *C* from the 10 μs simulations either considering all heavy or only Cα atoms of the protein (in both cases after we superposed the protein using the Cα coordinates). In the case of Ubq, we discarded the six C-terminal residues from our analyses, since they belong to a relatively unstructured C-terminal tail, whose motions might otherwise hide important differences in the remainder of the protein.

As some of our analyses aimed to compare the ensembles obtained with different force fields, we used, for those comparisons, a single, common essential subspace derived from a PCA of the entire set of simulations (one separate analysis for each of the two proteins). For the analyses involving individual proteins only, we performed the PCA analysis of the trajectories individually.

When representing a set of protein ensembles in a subspace of the (most important) PCs, the Root Mean Square Inner Product (RMSIP) is a natural measure of the similarity between the regions of conformational space sampled by different trajectories, and here we used the subspace described by the first 10 eigenvectors from the PCA to calculate the RMSIP [11].To quantify the extent to which the observed differences between two simulations (*A* and *B*) could be explained by the lack of convergence within the simulations rather than reflecting a genuine difference, we calculated the RMSIP between the first and second half of each simulation as a baseline. Calling the RMSIP between two simulations *RMSIP*_*AB*_ and the RMSIP between the first and second half of simulation *A*, *RMSIP*_*A1A2*_, we thus calculated a “normalized” score using ${RMSIP}_{AB}/\sqrt{{RMSIP}_{A1A2}{RMSIP}_{B1B2}}$. By normalizing *RMSIP*_*AB*_ with the “self-similarities” within the two simulations we expect to highlight differences between two simulations that go beyond what can be explained by sampling alone. Thus, unity values of this normalized RMSIP indicate that the similarity between the two simulations is of the same magnitude as that between the two halves of each simulation. In contrast, lower values indicate that differences between the two simulations are greater than what is explained by sampling alone.

## Results and Discussion

### Evaluation of MD force fields in the essential subspace

**Fig 1:**
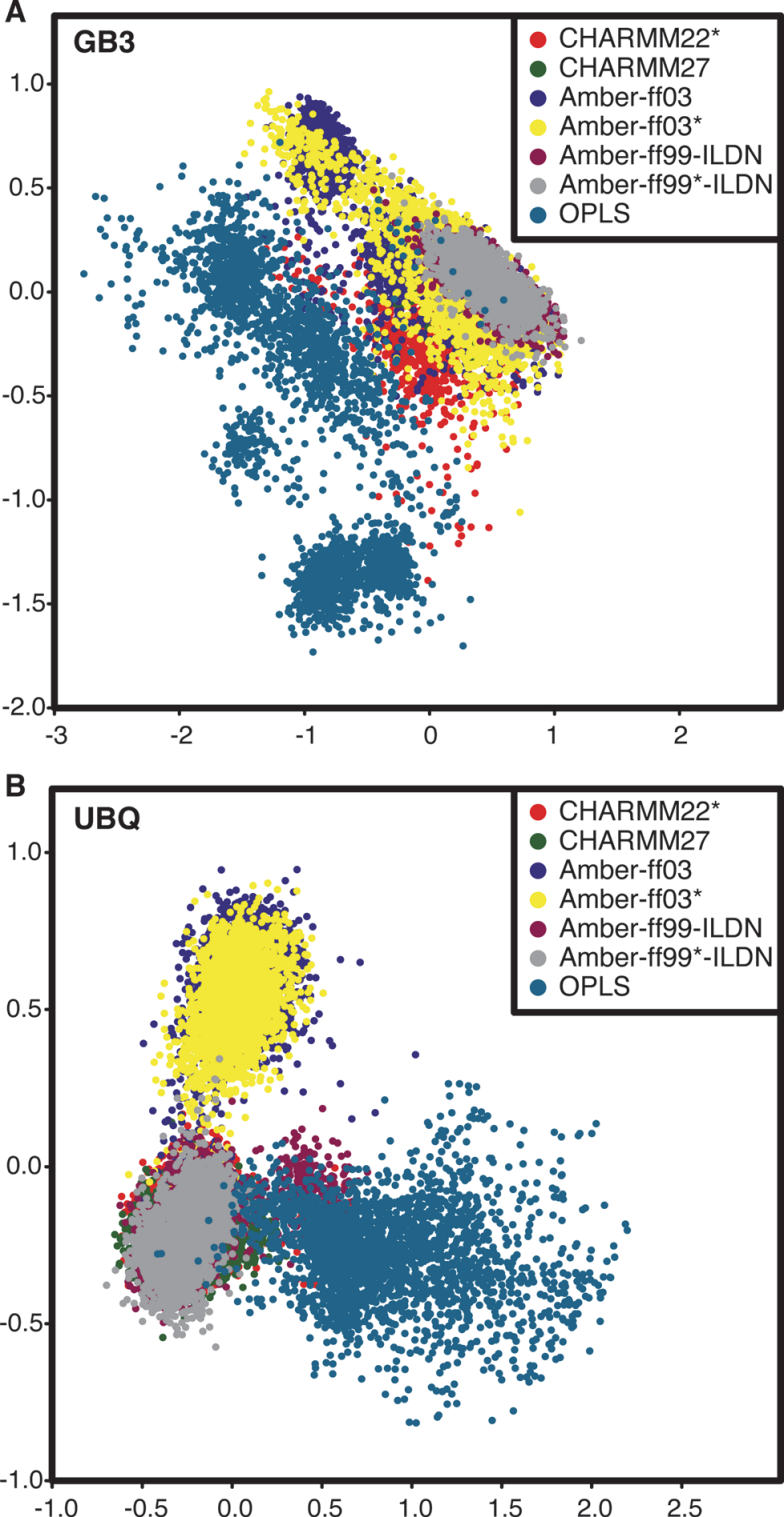
Comparison of force fields in the essential subspace. The figure shows the distributions of conformations of (A) GB3 and (B) Ubq projected on to the first two principal components. The PCA shown here includes seven of the eight force fields that we studied, with CHARMM22 omitted from the analysis for sake of clarity (see also main text and S1 and S2 Figs.).

We performed a PCA of the simulations of Ubq and GB3 to provide an overview and comparison of the structural ensembles obtained with the eight different force fields and to complement the previously published comparison to experimental data [4]. Initially, we analysed all eight force fields simultaneously. This analysis revealed, however, that the ensembles generated by the CHARMM22 force field sampled a much larger region of conformational space than the remaining simulations (S1 Fig.). To increase the level of resolution available in our analysis we thus repeated the PCA excluding the CHARMM22 force field (Fig. 1 and S2 Fig.). These analyses show that the ensembles fall, roughly, into three different classes. First, the Amber ff99SB-ILDN, Amber ff99SB*-ILDN, CHARMM22* and CHARMM27 force fields all seem to cover relatively similar and well-defined structural ensembles. Second, the Amber ff03 and ff03* ensembles cover a distinct, yet also relatively narrow structural ensemble. Finally, the OPLS ensemble, and more dramatically the CHARMM22 ensemble, covers a broad range of structures that only partially overlap with those sampled with the other force fields, and in the case of simulations of GB3 with CHARMM22 unfolding is eventually observed.

These three classes mirror the behaviour observed previously in the comparison to a broad range of NMR data, thus providing the structural basis of these results [4]. In particular, it becomes clear that the worse agreement between experiment and simulations for ff03 and ff03* (compared to the four force fields in the first class) is not due to excessive structural drift, but instead that these ensembles occupy a relatively well defined region of conformational space that is slightly shifted from the region spanned by the more accurate simulations with the four force fields in the first class (Fig. 1 and S2 Fig.). This observation also provides a demonstration of the kind of information that can more readily be extracted from a PCA than simply from calculating the RMSD to a specific reference structure. In particular, while the RMSD to the NMR structure is similar for both the ff03 and ff03* simulations, the PCA provides much stronger evidence that the two ensembles are very similar, and distinct from the first class. In contrast, the decreased agreement between experiments and simulations for OPLS and CHARMM22 is related to a more substantial drift of conformations away from the native structure during the simulation. This behaviour is supported further by inspection of the distribution of the projection on to the first PC (S3 Fig.). In particular, we find more complicated and multimodal distributions of the first PC for the ensembles that deviate the most from the experimental data and, in several cases, narrower and unimodal distributions for the remaining ensembles.

**Fig 2:**
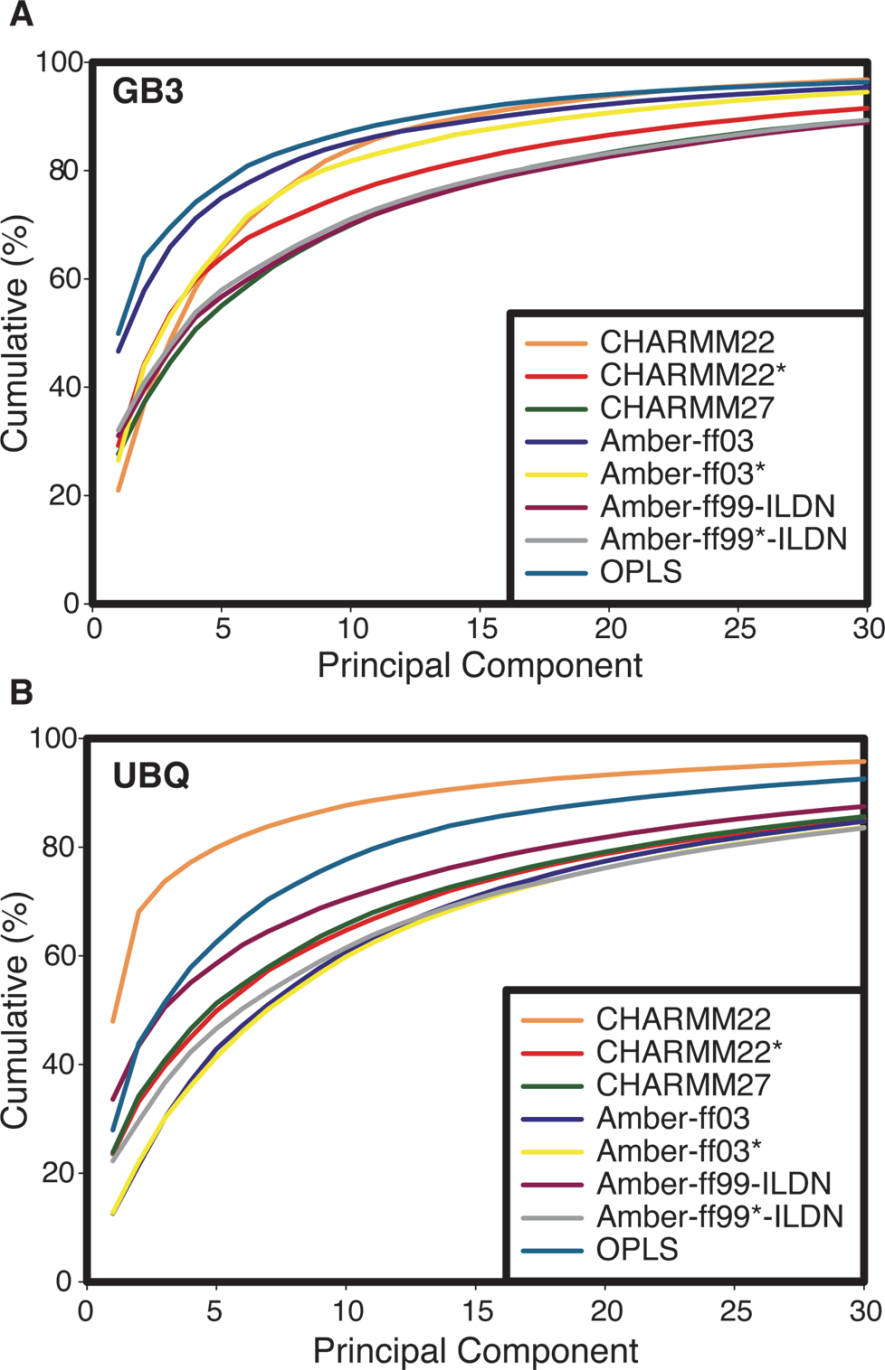
Different simulations require different numbers of PCs to capture the conformational heterogeneity. The figure shows the cumulative sums of the variance described by the principal components in the (A) GB3 and (B) Ubq simulations with eight different force fields. Note the large spread in the number of PCs that are required to capture the variability observed in the simulations, with between ~6 and ~25 PCs required to explain 80% of the variability.

Major conformational changes between a few distinct conformational states are expected to be reasonably well described by projections onto the first few principal components. In contrast, the dynamics of a protein within a single energetic well may involve independent motions of many different parts of the protein, and hence inherently have a higher (local) dimensionality. As the native states of both Ubq and GB3 are known experimentally mostly to undergo relatively small conformational fluctuations we suspected, as outlined above, that larger scale conformational changes occurring on a 10-μs timescale might be suggestive of a deterioration of the correct, folded structure rather than accurately representing large scale dynamics in these proteins. In the context of PCA, the dimensionality of the system manifests itself in the number of PCs that are needed to describe a certain fraction of the variability observed. To examine whether we can identify any correlation between the agreement of the simulations with experimental data and the range of conformational states observed, we calculated the fraction of the variation explained by the PCs as a function of the number of PCs included in this analysis (Fig. 2). In Ubq, we find that for the CHARMM22 and OPLS ensembles more of the observed variation can be explained by the first few eigenvectors, indeed suggesting that the structural dynamics observed for Ubq with these force fields can be captured in a lower dimensional space. The same observation is true in the case of OPLS and ff03 for GB3 (Fig. 2), with the latter also characterized by a bimodal distribution of the first PC (S3 Fig.). As these ensembles also are in less good agreement with experiments we suspect that the apparent lower dimensionality observed is an artefact of structural drift rather than an inherent property of the dynamics in the two proteins we studied. Indeed, it has previously been pointed out that care should be exerted in distinguishing structural dynamics from conformational drift when performing PCAs of molecular simulations [12,14]. Although it is particularly appealing to examine only the first few PCs (such as through two-dimensional projections) our results suggest that, at least for relatively rigid systems, the dynamics is of intrinsically higher dimensionality and that substantial (and possibly important) motions are hidden in dimensions beyond the first few. Indeed, the so-called “sketch-map” scheme for representing conformational space [17] is based on the finding that the effective dimensionality is high on short length-scales (individual conformational basins)but lower on longer length scales (for example involving transitions between distinct basins).

**Fig 3:**
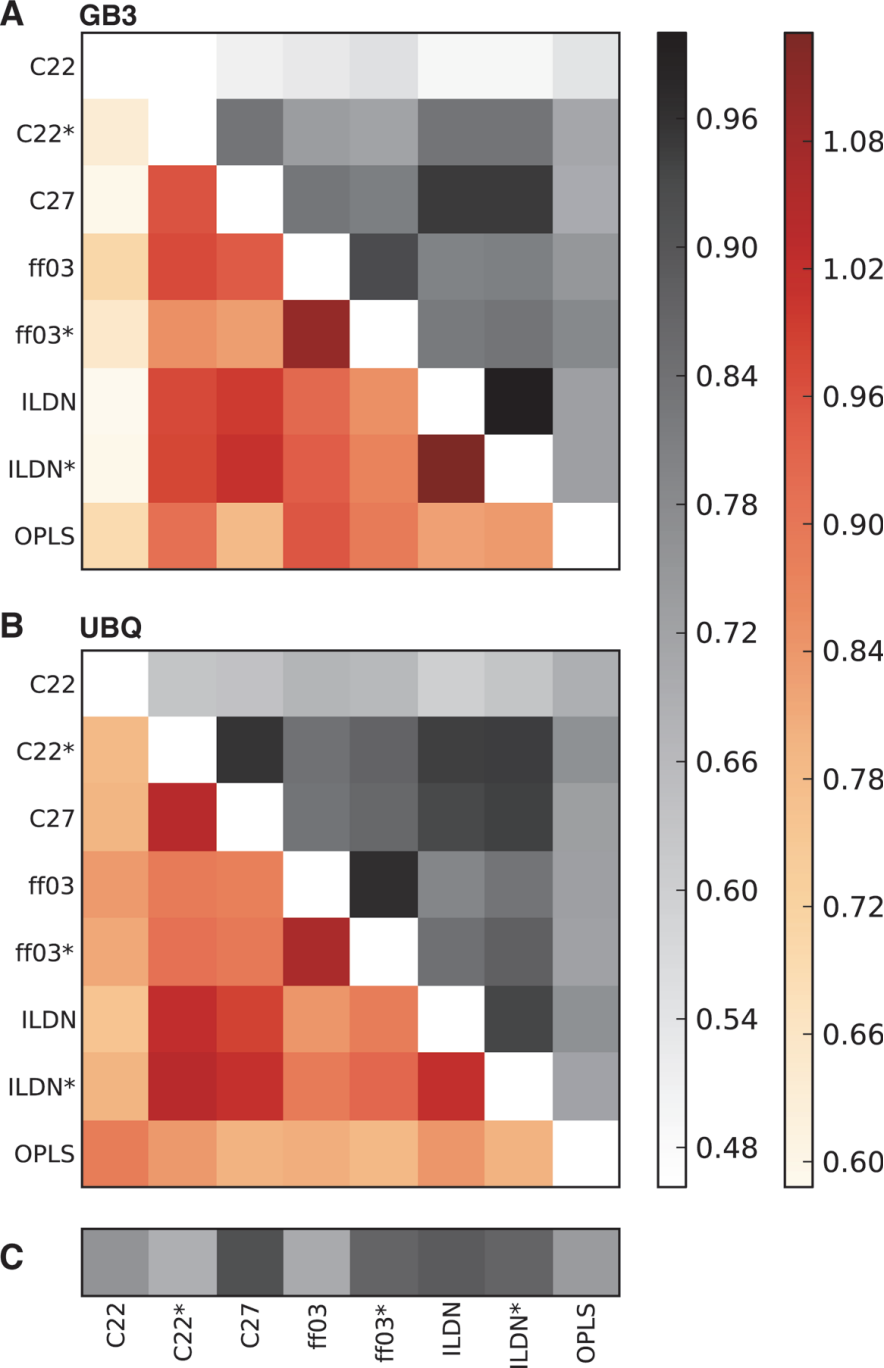
Quantifying the similarity of the conformational spaces captured in the different simulations. The figure shows a matrix of the pairwise similarities, quantified by the RMSIP, between simulations of (A) GB3 and (B) Ubq with eight different force fields. The upper triangular matrix (different shades of grey) reports the pairwise *RMSIP*_*AB*_ values between the different simulations. The lower triangular matrix (different shades of red) shows the values corresponding to a normalized RMSIP-value, calculated as ${RMSIP}_{AB}/\sqrt{{RMSIP}_{A1A2}{RMSIP}_{B1B2}}$, where *RMSIP*_*A1A2*_ and *RMSIP*_*B1B2*_ correspond to the RMSIP between the two halves of each simulation. C) The RMSIP values between the two halves of each simulation. Certain pairs of force fields (e.g. ff99SB-ILDN and ff99SB*-ILDN) give rise to very similar conformational sampling despite having substantially different propensities to form local helical structures.

In order to quantify the overlap between the different regions of the conformational space sampled in simulations performed with different force fields, also taking into account any differences and similarities beyond the first two dimensions, we calculated the RMSIP between all pairs of simulations for each of the two proteins (Fig. 3; above the diagonal). The RMSIP is a measure of the similarity of the structural space described by a set of PCs with a value of unity indicating identical subspaces and lower values indicating various levels of differences. Our results, using the first 10 PCs to calculate the RMSIPs, are fully consistent with the results obtained from inspection of the two-dimensional projections described above. In particular, at the highest level the CHARMM27, CHARMM22*, ff99SB-ILDN and ff99SB*-ILDN ensembles are relatively similar, while the OPLS and CHARMM22 are distinctly different. At a finer level of detail we find that the ff99SB-ILDN and ff99SB*-ILDN ensembles are very similar, as are the ff03 and ff03* ensembles, again in good agreement with the analysis in two dimensions.

A quantitative analysis of the RMSIP-values between two simulations A and B (*RMSIP*_*AB*_) is hampered by the fact that values less than unity may also arise from inadequate sampling in the two simulations rather than inherent differences in the two force fields. In an attempt to quantify the influence of sampling on the calculated values, we also calculated RMSIP between the first and second half of each simulation (horizontal bars in Fig. 3). These values, which we term *RMSIP*_*A1A2*_ (for simulation A), provide a rough baseline for any differences that are explained by sampling alone. We therefore calculated a “normalized” RMSIP value taking this factor into account using ${RMSIP}_{AB}/\sqrt{{RMSIP}_{A1A2}{RMSIP}_{B1B2}}$ (Fig. 3; below the diagonal). Here values of unity indicate that the similarity between the two simulations is as great as that between the first and second half of each simulation, whereas a value less than unity indicates that the differences between the two simulations is greater than what is easily explained by sampling alone. We find that several pairs of force fields give rise to ensembles that are essentially identical within “error” as defined by this metric, but also find that the differences between certain pairs of ensembles (e.g. comparing the ff99SB-ILDN/ff99SB*-ILDN with ff03/ff03*) are greater than what can easily be explained by sampling.

Calculation of RMSIP between the first and second half of a simulation is a frequently used approach to evaluate to what extent a simulation continues to sample new conformations (low RMSIP values) or whether the same structures are recurring. In this kind of analyses, RMSIP values higher than 0.6–0.7, are generally employed as a threshold [14–15]. In this respect, it is interesting to note that we find that simulations carried out with different force fields, which have different agreement with the experimental data and sample different structural ensembles, (e.g. OPLS vs ff99SB*-ILDN), are still characterized by similar RMSIP values around 0.65, suggesting that determination of convergence based on RMSIP values alone can give misleading results. Indeed, an alternative test for convergence not only gauges which structures are sampled, but also quantifies the extent to which the populations of these structures can robustly be determined [18].

### Timescales of motions in ubiquitin and GB3

**Fig 4:**
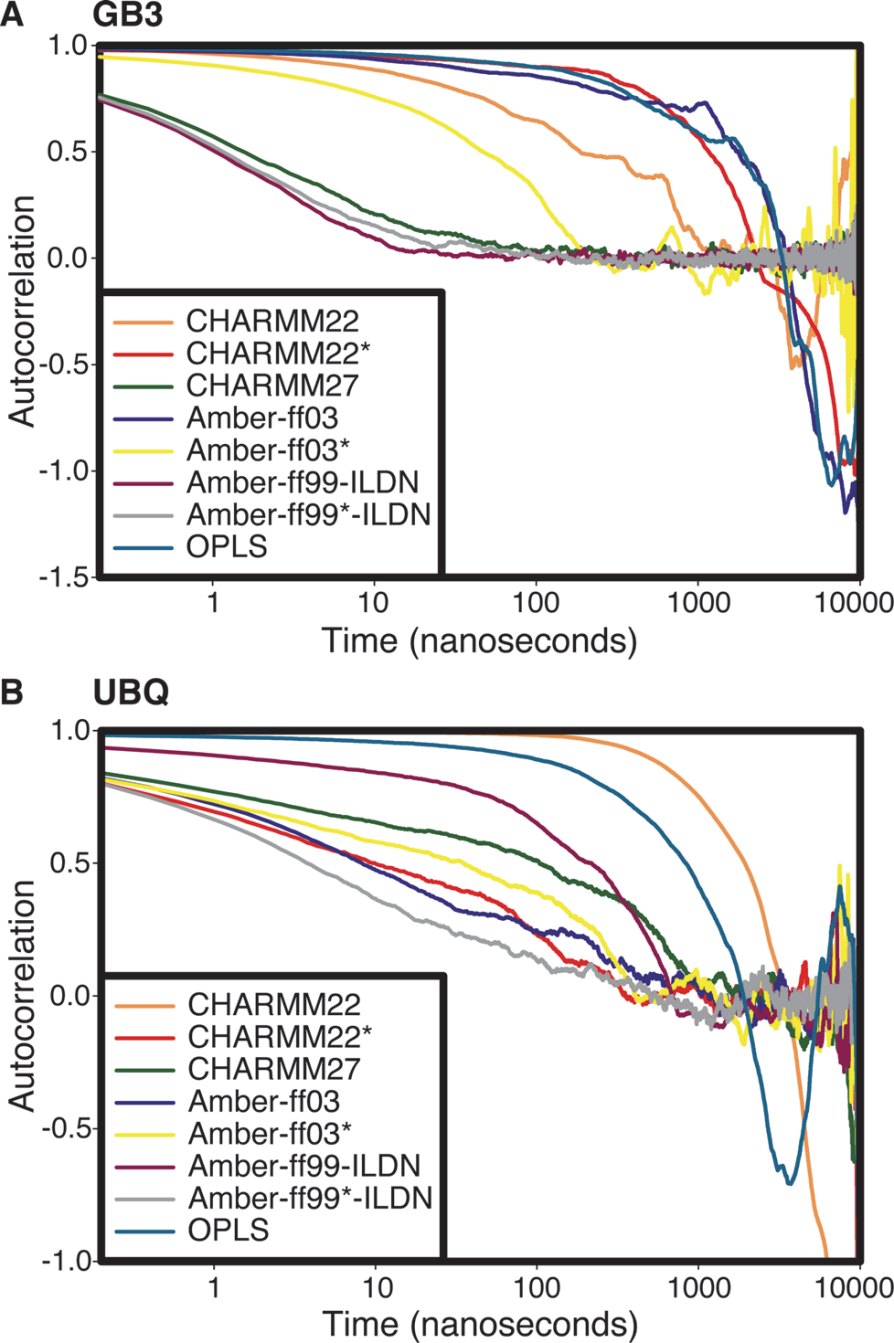
Analysis of the timescales of motions along the first principal component. The figure shows the autocorrelation function of the values of first principal component from the eight simulations of (A) GB3 and (B) Ubq. As the lag-times approach the duration of the simulation, the calculated values become increasingly uncertain, but it is still clear that the different simulations display dynamics on quite different time scales (note the logarithmic time axis).

As a means to estimate the timescales associated with the dominant motions observed in the simulations, and an alternative to evaluate the convergence of the simulations using the PCA framework, we calculated the autocorrelation function (ACF) of the first PC for each of the simulations (Fig. 4). In general, the results confirm the observations above: the force fields that give the most accurate description of the two proteins have faster relaxation along the dominant PC, whereas the other force fields relax more slowly—presumably an indication of the structural drift observed in at least some of these simulations. The analysis, however, also reveals relatively slow relaxations in simulations of GB3 with CHARMM22* and ff03. As discussed in more detail in the next section, this observation could in principle be due to at least two different scenarios: (i) a structural drift or (ii) conformational changes between a few distinctly different substates occurring on a longer timescale.

### Structural comparison

**Fig 5:**
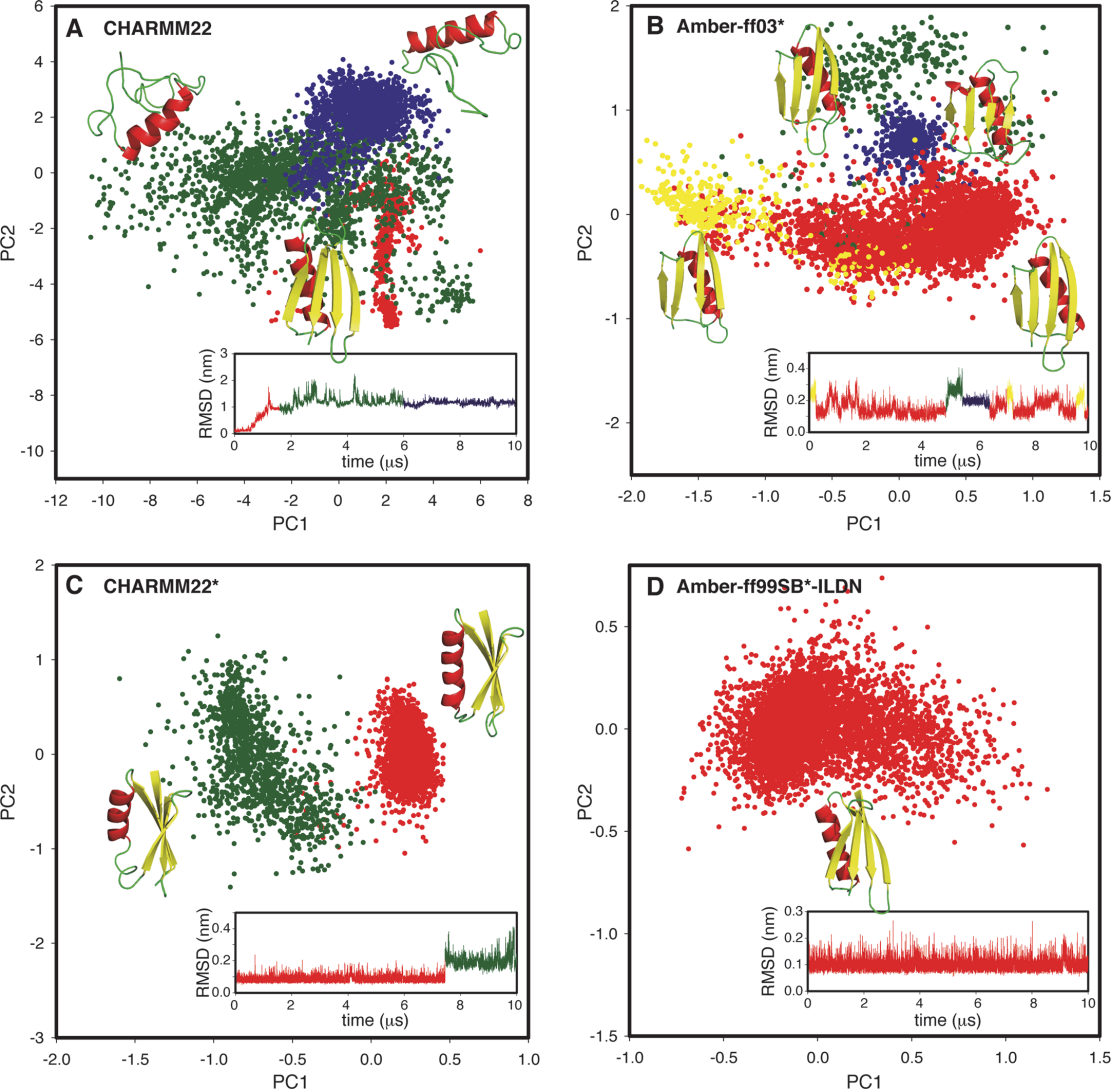
A wide variety of conformational sampling is observed in the simulations. The figure shows four examples of different kinds of conformational sampling observed in the simulations. The simulations shown here are of GB3 with (A) CHARMM22, (B), ff03*, (C) CHARMM22* and (D) ff99SB*-ILDN, with the entire set of simulations shown in S4 and S5 Figs. Briefly, (A) exemplifies a simulation with a large conformational drift, (B) is a simulation with (partially) reversible sampling of different substates, (C) is a simulation with an irreversible sampling of a second substate and (D) is a simulation that is fully stable in the initial starting state for the entire duration of the simulation. Note that eventually all force fields are expected to sample different states, but with rates of transitions and stabilities of the different states highly dependent on the chosen force field.

To provide a clearer picture of how the proteins behave when different force fields are used, we analysed the structures explored in the different simulations using the two-dimensional projections along the first and the second PCs (Fig. 5 shows four representative examples for GB3; S4 and S5 Figs. contain all simulations). Each of the four examples presented in Fig. 5 represents a distinct scenario observed in these simulations. The first class is here exemplified by CHARMM22 (Fig. 5A) and also includes OPLS. In these simulations the structure relatively quickly moves away from the native conformation, showing major rearrangements in particular in the β-sheet and loops in the two proteins. The drift begins within the first few hundred ns and continues for the remainder of the simulation time. In the simulation of GB3 with CHARMM22 the effect is particularly clear with an unfolding of the protein with the exception of the helical region (Fig. 5A). These simulations are thus clearly not converged, and the fact that the first PCs account for the majority of their dynamical features more likely reflects the general drift away from the native structure, which is also consistent with these force fields having a lower agreement with the experimental data.

A second class, here exemplified by the simulation of GB3 with ff03* (Fig. 5B), samples structures that are similar to the experimentally derived structures (red), but also (transiently) includes structures that differ more from the starting structure (yellow, blue and green).The simulation quickly moves away from the initial structure, but returns closer to it later in the trajectory. The structural substates that differ from the initial structure are characterized by subtle changes in the parallel β-sheet region or the C-terminal helix of GB3, and thus cannot be ascribed to a general drift away from the experimental structure or to unfolding events. In this class, we also find the simulation of Ubq with ff99SB-ILDN, though the excursions observed in this simulation are much more limited (accounting for about 7% of the frames) and are structurally restricted to a small change in the conformation in the C-terminus of the α-helix.

The third scenario is exemplified by the simulations of both GB3 and Ubq with ff03, of Ubq with ff03* and CHARMM27, and of GB3 with CHARMM22* (Fig. 5C, and S4 and S5 Figs.). In all of these simulations the protein initially populates a single region of conformational space, but later transitions to a different, also relatively well-defined state without revisiting the original state within the 10 μs simulation. The ff03 and Ubq ff03* trajectories distinguish themselves within this class by making their transitions relatively early, and then populate a single state for the remainder of the simulation. The simulations of GB3 with CHARMM22* and of Ubq with CHARMM27 have the opposite behaviour, as they stably sample the conformational region corresponding to the experimentally determined structure for most of the time, but structural changes from this main region are observed after 7 and 9 μs, respectively. It is worth noting that a longer, millisecond-length MD simulation of Ubq with CHARMM22* [19] showed several reversible structural transitions of this kind. Indeed, in that simulation 10% of the structures were characterized by a transient loss of secondary structure at the C-terminal part of the α-helix, a motion that might be present in nature but overemphasized by the force field [19].

The fourth case is represented by the force fields that continuously sample structures very similar to the initial, NMR structures (Fig. 5D). These are also the force fields for which the simulations cannot be described well by a small number of PCs (Fig. 2), and which generally feature fast relaxation along the first PC (Fig. 4). This scenario is exemplified by the simulation of GB3 with ff99SB*-ILDN (Fig. 5D), and is also observed in simulations of GB3 with CHARMM27 and ff99SB-ILDN, as well as of Ubq with ff99SB*-IDLN.

We stress that the classification of a simulation into one of the classes above depends not only on force-field quality, but also on the length of the simulation as well as the stochastic nature of molecular simulations. Thus, our results highlight the difficulty of achieving converged results in molecular simulations. Proteins that are fully stable in the 10 μs simulation analysed here (class 4) would likely later transition into other states (class 3) and return to the initial state even later (class 2). Alternatively, the simulation can continue to explore new states that deviate even more from the initial structure (class 1). Of course, whether a structural transition occurs in a given simulation time (either in a single trajectory or distributed across multiple simulations) also depends on the stochasticity of the system. If a simulation eventually reaches the timescales of unfolding and folding, which are likely the longest timescales present in most proteins, one would expect to observe reversible transitions between fully folded and unfolded structures. Comparisons to experimental measurements on folding kinetics and thermodynamics from such converged simulations provide rather stringent tests of force field quality exactly because they presumably sample the longest timescales of conformational changes [20,21]. So far, however, this has mostly been possible for the fastest folding proteins and with simulations substantially longer than those analysed here.

The projections by PCA of the conformational substates also highlight how the RMSD to a specific reference structure cannot always capture the complexity of the free energy landscape sampled in a simulation. In particular, there are several examples, in particular in GB3 (S4 Fig.), where distinct states (as revealed by PCA) have similar RMSD-values to the NMR structure. Thus, while an RMSD-calculation is often the most natural and easily performed analysis, projection methods can substantially aid in finding and characterizing differences between simulations—for instance arising from the use of different force fields.

**Fig 6:**
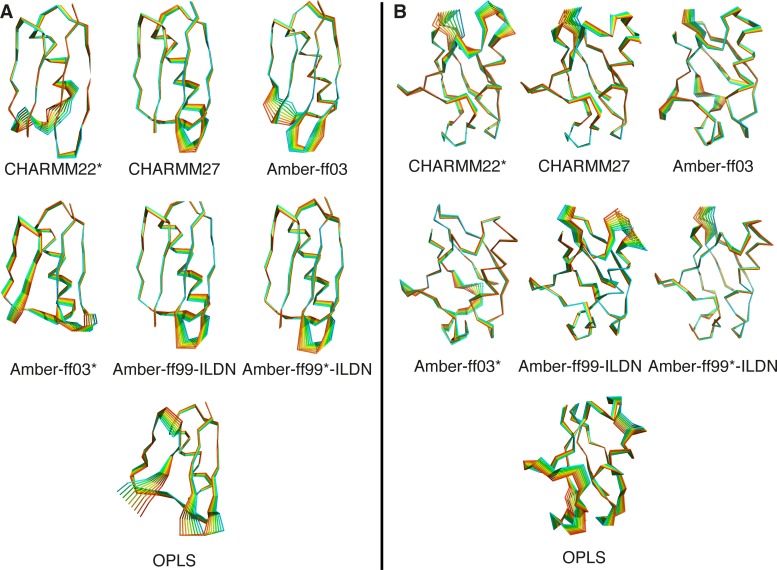
Dominant motions observed in simulations with different force fields. The figure shows a structural representation of the global motions in (left) GB3 and (right) Ubq that are captured in the first three principal components, scaling each by the associated eigenvalue. We extracted 10 structures from this analysis for each simulation (excluding CHARMM22 because of the large conformational drift) and coloured these from blue to red to help visualize the regions of the proteins that display larger scale motions.

Finally, to visualize the motions observed in the different simulations and to compare the information provided by the first three PCs of each simulation, we mapped the largest amplitude motions of each simulation (excluding those with CHARMM22) on to the structures of GB3 and Ubq (Fig. 6). These global motions are the result of the combination of the coordinates of the first three PCs, scaling them by their associated eigenvalues. Interestingly, we find that several of the force fields produce larger motions in similar regions, in particular loops, but that the amplitude of these motions generally differ.

## Conclusions

We present a comparison of eight different force fields (CHARMM22, CHARMM22*, CHARMM27, Amber ff03, Amber ff03*, Amber ff99SB-ILDN, Amber ff99SB*-ILDN and OPLS) in the essential subspace described by a PCA of 10 μs MD simulations of Ubq and GB3. Together with a previously published comparison with experimental data, our study provides an overall view of the effects that different force-field parameterizations can have on the structural ensemble obtained from MD simulations.

At a coarse level, our results show that many aspects of the larger scale motions are rather well preserved across several force fields, with the only exceptions being CHARMM22 and to some extent OPLS. This observation suggests that MD simulations carried out with recent and experimentally validated force fields can robustly describe the overall dynamics of a protein. Presumably, motions conserved across different simulations with distinct force fields are more likely to represent nature, though such a “consensus” approach to simulations should be tested more directly in future studies.

In addition, we observe that simulations with the Amber force fields that have been reparameterized to provide a more accurate energetic description of the balance between helical and coil structures (ff99SB*-ILDN and ff03*) are difficult to distinguish from their “unbalanced” counterparts (ff99SB-ILDN and ff03, respectively). This observation implies that simulations of stable, folded proteins, even those reaching 10 μs in length, cannot always provide information to modify the torsion parameters related to the secondary structural elements, and that such reparameterization should preferably also include simulations of flexible peptides that sample the conformational space more comprehensively. Recently, updated versions of both the CHARMM and OPLS force fields have been developed [22–24] and it will be interesting to see the results of similarly long simulations of Ubq and GB3 with these force fields in the context of the PCA performed here.

At a finer level of detail, our results also show that there are distinct differences between the force fields. Indeed, some of the force fields result in sampling of conformational substates whose properties and populations are incompatible with the experimental NMR data. It is important to note, however, that even 10 μs simulations on fairly rigid systems only reveal some of such differences. In particular, in the case of Ubq, a millisecond simulation has revealed transitions not observed in the shorter 10 μs simulations analysed here [19]. Indeed, many proteins are known to undergo conformational changes on the microsecond to millisecond time scale and in this way sample sparsely populated states, though validating the observed states experimentally may be difficult.

Even when sparsely populated states are observed in simulations, their relative populations are difficult to estimate reliably from a simulation. In a previous benchmark study of several force fields that used 1 μs long simulations, the authors concluded that it was hard to define if the force fields needed to be corrected to destabilize the rare conformational states, or whether longer simulations times were required to allow the relative weight of these states to converge [7]. In this context, we show that even on a time scale one order of magnitude longer (1 μs vs. 10 μs), we are still not able, with the currently employed force fields, to provide a distribution of the rare conformational states comparable to the population observed experimentally. This is also true for millisecond-length simulations of Ubq [19] and BPTI [25], though a recent application of an enhanced sampling method allowed for an accurate determination of a conformational free energy change in Cyclophilin A [26].

PCA is often used as a tool to achieve a compact, more readily interpreted description of protein dynamics. Nevertheless, we here observed that—even in the case of well folded, small and relatively rigid proteins—simulations with even the most accurate force fields do still not always converge in the context of the reductive description provided by PCA. The dynamics of small, single-domain proteins such as Ubq and GB3 often consists of subtle, often relatively independent motions of many different part of the protein and is therefore inherently of high dimension. It should therefore be considered a potential sign of warning when a PCA of such proteins suggests that most of the dynamics can be described by a very small number of principal components, unless these correspond to larger, distinct motions. For larger conformational changes such as transitions between alternative conformations or independent motions of protein domains, the longer length scale motions may well be expected to be of lower dimensionality, while the dynamics within the individual basins might be of higher dimensionality. As we observe here for simulations of the native state of the two relatively rigid proteins Ubq and GB3, the finding of a few dominant PC’s is in these cases a signal of the deterioration of the folded structure due to force field deficiencies rather than large amplitude motions of functional relevance. We note, though, that on longer timescales or in more complex systems, the results may differ. In all cases, a tighter integration of PCA of simulations with different force fields and the validation against experimental data can provide insight into the dynamical properties of proteins in atomistic details.

## Supporting Information

S1 Fig Comparison of force fields in the essential subspace. The figure shows the distributions of conformations of (A) GB3 and (B) Ubq projected along the first two principal components. The results shown here are of a PCA of the simulations with all eight force fields that we studied, and can be compared to Fig. 1 in the main text, which excluded CHARMM22 from the PCA.
(DOCX)

S2 Fig Comparison of force fields in the essential subspace without CHARMM22. The figure shows the distribution of conformations of (A) GB3 and (B) Ubq projected along the first two principal components. The PCA shown here includes seven of the eight force fields that we studied, with CHARMM22 omitted from the analysis for sake of clarity (see also main text). In particular, we show here the same results presented in Fig. 1 with each force field in a separate panel.
(DOCX)

S3 Fig Distribution of the projection onto the first principal component. The plot shows the distribution of values when projected onto the first principal component for (upper) GB3 and (lower) Ubq. Some force fields show simple, unimodal distributions and others more complex distributions. As discussed in the main text, multimodal behaviour is in some, but not all, cases the result of a conformational drift in the simulations.
(DOCX)

S4 Fig Substates explored in the simulations of GB3 with different of force fields. In the plots, each point represents a conformation of the ensemble sampled with the given force field and are shown along the first two principal components in a PCA of that simulation. The points have been colour-coded according to the region of the PCA subspace that they populate to ease visualization. The inserts show a time series of the RMSD to a reference structure, colour-coded in the same way, so that the temporal progression of the sampling can be assessed.
(DOCX)

S5 Fig Substates explored in the simulations of Ubq with different of force fields. In the plots, each point represents a conformation of the ensemble sampled with the given force field and are shown along the first two principal components in a PCA of that simulation. The points have been colour-coded according to the region of the PCA subspace that they populate to ease visualization. The inserts show a time series of the RMSD to a reference structure, colour-coded in the same way, so that the temporal progression of the sampling can be assessed.
(DOCX)
