# Supplementary material for: Comparing Molecular Dynamics Force Fields in the Essential Subspace
Source: PLoS One. 2015 Mar 26;10(3):e0121114. doi: 10.1371/journal.pone.0121114 (PMC4374674; doi:10.1371/journal.pone.0121114)
Supplement: S1 Fig — The figure shows the distributions of conformations of (A) GB3 and (B) Ubq projected along the first two principal components. The results shown here are of a PCA of the simulations with all eight force fields that we studied, and can be compared to Fig. 1 in the main text, which excluded CHARMM22 from the PCA. (DOCX) [file pone.0121114.s001.docx]

**S1 Fig. Comparison of force fields in the essential subspace.** The figure shows the distributions of conformations of (A) GB3 and (B) Ubq projected along the first two principal components. The results shown here are of a PCA of the simulations with all eight force fields that we studied, and can be compared to the Fig.1 in the main text, which excluded CHARMM22 from the PCA.

**
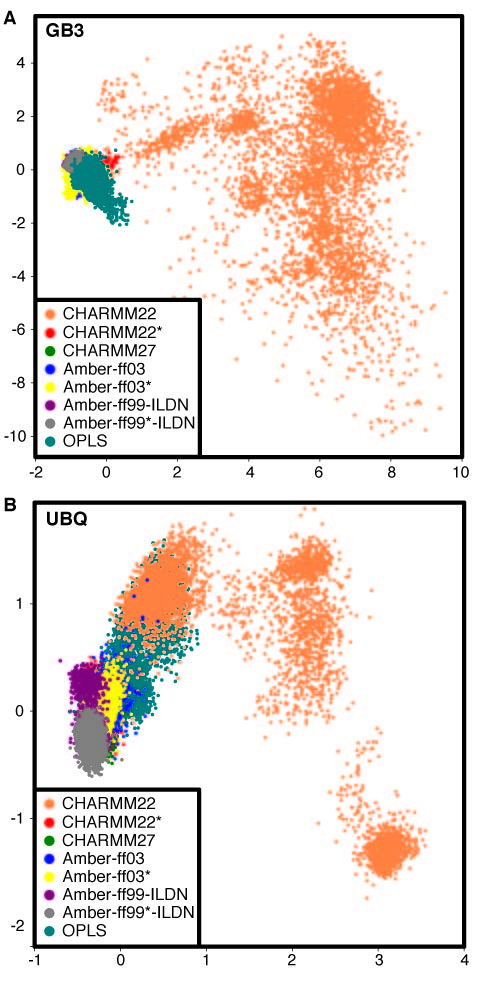
**
