# Supplementary material for: Comparing Molecular Dynamics Force Fields in the Essential Subspace
Source: PLoS One. 2015 Mar 26;10(3):e0121114. doi: 10.1371/journal.pone.0121114 (PMC4374674; doi:10.1371/journal.pone.0121114)
Supplement: S2 Fig — The figure shows the distribution of conformations of (A) GB3 and (B) Ubq projected along the first two principal components. The PCA shown here includes seven of the eight force fields that we studied, with CHARMM22 omitted from the analysis for sake of clarity (see also main text). In particular, we show here the same results presented in Fig. 1 with each force field in a separate panel. (DOCX) [file pone.0121114.s002.docx]

**S2. Fig. Comparison of force fields in the essential subspace without CHARMM22.**The figure shows the distribution of conformations of (A) GB3 and (B) Ubq projected along the first two principal components. The PCA shown here include seven of the eight force fields that we studied, with CHARMM22 omitted from the analysis for sake of clarity (see also main text). In particular, we show here the same results presented in Figure 1 with each force field in a separate panel.

**A**

**B**
