# Supplementary material for: Comparing Molecular Dynamics Force Fields in the Essential Subspace
Source: PLoS One. 2015 Mar 26;10(3):e0121114. doi: 10.1371/journal.pone.0121114 (PMC4374674; doi:10.1371/journal.pone.0121114)

**S3 Fig. Distribution of the projection onto the first principal component.** The plot shows the distribution of values when projecting onto the first principal component for (upper) GB3 and (lower) Ubq. Some force fields show simple, unimodal distributions and others more complex distributions. As discussed in the main text, multimodal behaviour is in some, but not all, cases the result of a conformational drift in the simulations.


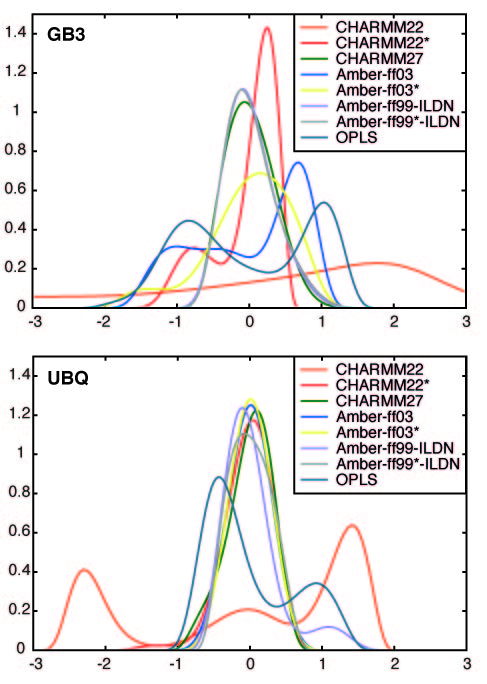

Supplement: S3 Fig — The plot shows the distribution of values when projected onto the first principal component for (upper) GB3 and (lower) Ubq. Some force fields show simple, unimodal distributions and others more complex distributions. As discussed in the main text, multimodal behaviour is in some, but not all, cases the result of a conformational drift in the simulations. (DOCX) [file pone.0121114.s003.docx]
