# Supplementary material for: Comparing Molecular Dynamics Force Fields in the Essential Subspace
Source: PLoS One. 2015 Mar 26;10(3):e0121114. doi: 10.1371/journal.pone.0121114 (PMC4374674; doi:10.1371/journal.pone.0121114)

**S4 Fig. Substates explored in the simulations of GB3 with different of force fields.** In the plots each point represents a conformation of the ensemble sampled with the given force field and are shown along the first two principal components in a PCA of that simulation. The points have been colour-coded according to the region of the PCA subspace that they populate to ease visualization. The insertsshow a time series of the RMSD to a reference structure, colour-coded in the same way, so that the temporal progression of the sampling can be assessed.


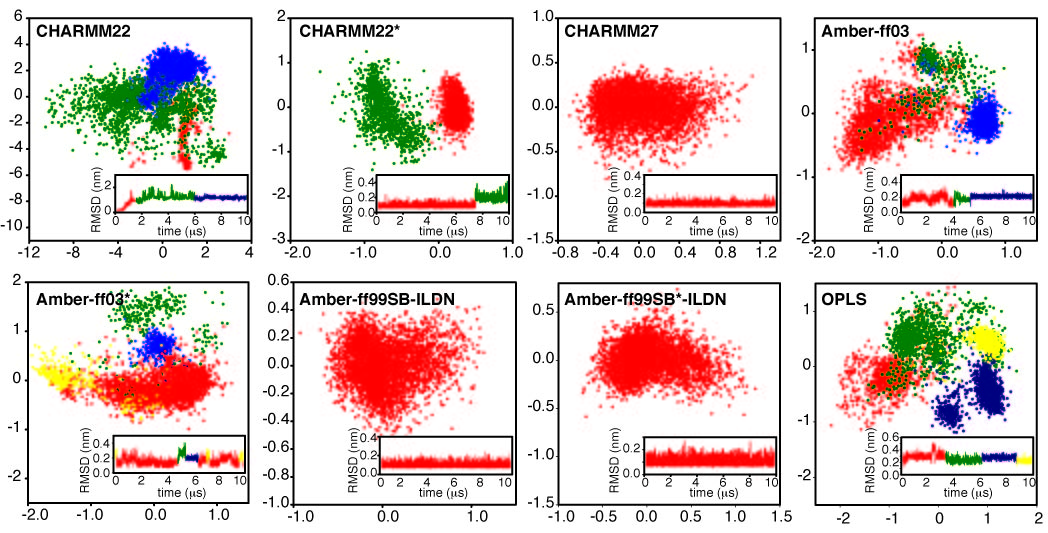

Supplement: S4 Fig — In the plots, each point represents a conformation of the ensemble sampled with the given force field and are shown along the first two principal components in a PCA of that simulation. The points have been colour-coded according to the region of the PCA subspace that they populate to ease visualization. The inserts show a time series of the RMSD to a reference structure, colour-coded in the same way, so that the temporal progression of the sampling can be assessed. (DOCX) [file pone.0121114.s004.docx]
